# Regionally adaptive physical training for university students considering environmental and social disparities

**DOI:** 10.3389/fpubh.2025.1679354

**Published:** 2025-10-02

**Authors:** Svetlana Kondratenko, Meruert Tuyakbaeva, Galiya Madiyeva, Saule Arkabaeva, Ali Zhalel, Natalia Shepetyuk

**Affiliations:** ^1^Department of Physical Education and Sports, Al-Farabi Kazakh National University, Almaty, Kazakhstan; ^2^Department of Physical Education and Sports, Kazakh National Agrarian Research University, Almaty, Kazakhstan; ^3^Department of Physical Education, Satbayev University, Almaty, Kazakhstan; ^4^Department of Physical Education and Sports, L.N. Gumilyov Eurasian National University, Astana, Kazakhstan

**Keywords:** polycyclic aromatic hydrocarbons, testosterone, density functional theory, molecular dynamics, air pollution, endocrine disruption, athletic performance, student athletes

## Abstract

Airborne polycyclic aromatic hydrocarbons (PAHs) are urban combustion by-products linked to endocrine disruption, but their direct molecular interactions with testosterone remain under-characterized. Using DFT [B3LYP/6–311 + G(d,p)] and 10-ns all-atom MD, we quantified non-covalent binding between benzene, naphthalene, and anthracene and testosterone, observing size-dependent stabilization (anthracene most favorable). Complementary MEP, Mulliken charge, and FMO analyses indicated progressive electronic coupling consistent with *π*–π and hydrophobic packing. In a semester-long controlled program with male university students (*n* = 60), we compared identical training conducted in a polluted urban area (PM_2.5_ > 50 μg·m^−3^) vs. a suburban green zone (PM_2.5_ < 10 μg·m^−3^) and observed larger gains in 100-m sprint, pull-ups, and standing long jump under cleaner air. We now report 95% confidence intervals alongside effect sizes for all field outcomes and provide a correlation between pollution intensity and performance change. PM_2.5_ was used as an operational exposure index because combustion-related PAHs predominantly partition to fine particles and co-vary with PM₂.₅ mass in ambient air (WHO guideline context and PAH–PM₂.₅ literature). Collectively, the molecular and field evidence suggests larger PAHs may perturb testosterone function and that cleaner air is associated with better short-term training gains, informing air-quality-aware scheduling and campus policy.

## 1 Introduction

Exposure to air pollution has emerged as a significant environmental and public health challenge, extending its impact beyond respiratory and cardiovascular systems to disrupt endocrine functions (1–3). Among atmospheric pollutants, polycyclic aromatic hydrocarbons (PAHs) are particularly concerning due to their persistent, lipophilic nature and known toxic effects. Generated predominantly through incomplete combustion of organic matter like fossil fuels and biomass, PAHs are notably prevalent in densely populated urban settings characterized by intensive traffic and industrial activities (4–7). Athletes, who frequently engage in outdoor training sessions during times of peak pollution, face heightened risks of inhaling these contaminants, potentially impairing respiratory efficiency, hormonal balance, and overall physiological well-being. Lower-limb explosive capacity is pivotal for sport performance—Countermovement Jump height tracks knee-extensor strength in elite boxers (8), and phase-specific diagnostics in elite men’s table tennis show that short, high-intensity “attacking/connecting” segments most strongly determine outcomes (9)—supporting our use of brief explosive tests (100-m sprint, standing long jump) as environmentally sensitive endpoints.

Operational exposure index (PM_2.5_). We used PM_2.5_ to operationalize “polluted” versus “clean-air” training days because many combustion-derived PAHs—particularly higher-ring species—partition to fine particles and consistently occur within the PM_2.5_ fraction; numerous urban field studies report particle-bound PAHs and co-variation with PM_2.5_ mass. While PM_2.5_ is non-specific to PAHs, it provides a practical exposure contrast for field protocols when speciated PAH monitoring is not available; thresholds in this study (>50 vs. < 10 μg·m^−3^) were selected to create a clear contrast relative to WHO guideline levels (10, 11).

Testosterone plays a critical role in regulating muscle growth, energy metabolism, mood stability, and reproductive health—factors directly influencing athletic performance and recovery (4, 12–17). Disruptions in testosterone dynamics, whether through altered biosynthesis pathways, receptor interactions, or degradation kinetics, can significantly compromise physical capabilities and lead to chronic health issues. Previous studies indicate that environmental exposure to PAHs correlates with changes in testosterone levels, though the molecular mechanisms underpinning these effects are still inadequately characterized. Specifically, there remains a significant gap in understanding at the atomic level how PAHs interact physically or electronically with testosterone molecules in biological contexts (12, 14, 17–21).

Prior research demonstrates a robust link between PAHs exposure and endocrine disruption relevant to athletic health. Studies confirm that PAHs can significantly alter testosterone concentrations in males, highlighting potential health risks associated with hormone imbalance (22). Mechanistic insights have further illustrated that PAHs influence hormonal regulation via disruptions in the hypothalamic–pituitary-gonadal (HPG) axis, affecting testosterone and estradiol levels variably, contingent upon specific PAH metabolites and individual demographic characteristics like age (23). Clinical evidence associates elevated estradiol and reduced testosterone levels with increased risks and severity of pulmonary arterial hypertension (PAH), underscoring broader systemic health implications stemming from hormonal imbalances (24).

Moreover, environmental endocrine disruptions have been linked to increased mortality in men, highlighting the urgency of addressing air pollutant exposures from both athletic and public health perspectives [19]. Investigations into broader air pollution constituents, including particulate matter (PM), ozone, and nitrogen dioxide, reveal detrimental impacts on athletes’ respiratory and cardiovascular functions, particularly during intense physical exertion (25). Endurance athletes frequently report reduced performance capacities and heightened susceptibility to health issues associated with air pollution exposure, emphasizing the necessity for ongoing research in this area (26). Practical assessments of athletes’ pollutant exposure during sports events indicate significant variability, further supporting the importance of monitoring and strategic timing to mitigate these risks (27).

Building upon these insights, the current study provides a detailed atomistic investigation into interactions between common PAHs and testosterone derivatives. To elucidate these interactions, we employed a multiscale computational methodology combining Density Functional Theory (DFT) and classical all-atom Molecular Dynamics (MD) simulations. DFT analysis enabled examination of electronic structural changes, dipole moments, and molecular orbitals of testosterone derivatives upon complexation with representative PAH molecules (naphthalene, phenanthrene, and benzo[a]pyrene). Complementary MD simulations assessed the dynamic behavior, solvation, and stability of these complexes within physiologically relevant aqueous environments.

Objectives and Hypotheses.

Our objective is to link atomistic interaction trends between PAHs and testosterone with observed training outcomes under contrasting ambient air-quality conditions. Accordingly, we prespecified three directional hypotheses that map directly to our analyses:

- H1 (molecular): Non-covalent association strength with testosterone will increase with PAH ring number—predicted order naphthalene < phenanthrene < benzo[a]pyrene—reflected by more favorable (more negative) binding energies and longer residence times in MD.
- H2 (field, between-group): University students training under lower PM₂.₅ conditions will exhibit larger short-term improvements in the 100-m sprint, pull-ups, and standing long jump than students training under higher PM_2.5_, after baseline adjustment.
- H3 (linking, within-group): Session-level PM₂.₅ will be negatively correlated with individual performance change scores across the same outcomes.

Primary outcomes are the three field tests listed above; exposure contrast is defined operationally by PM_2.5_ thresholds detailed in Methods. Analytical specifics (baseline-adjusted models, confidence intervals, and multiplicity control) are described in the Statistical Analysis subsection.

## 2 Materials and methods

### 2.1 Theoretical models

**Figure 1:**
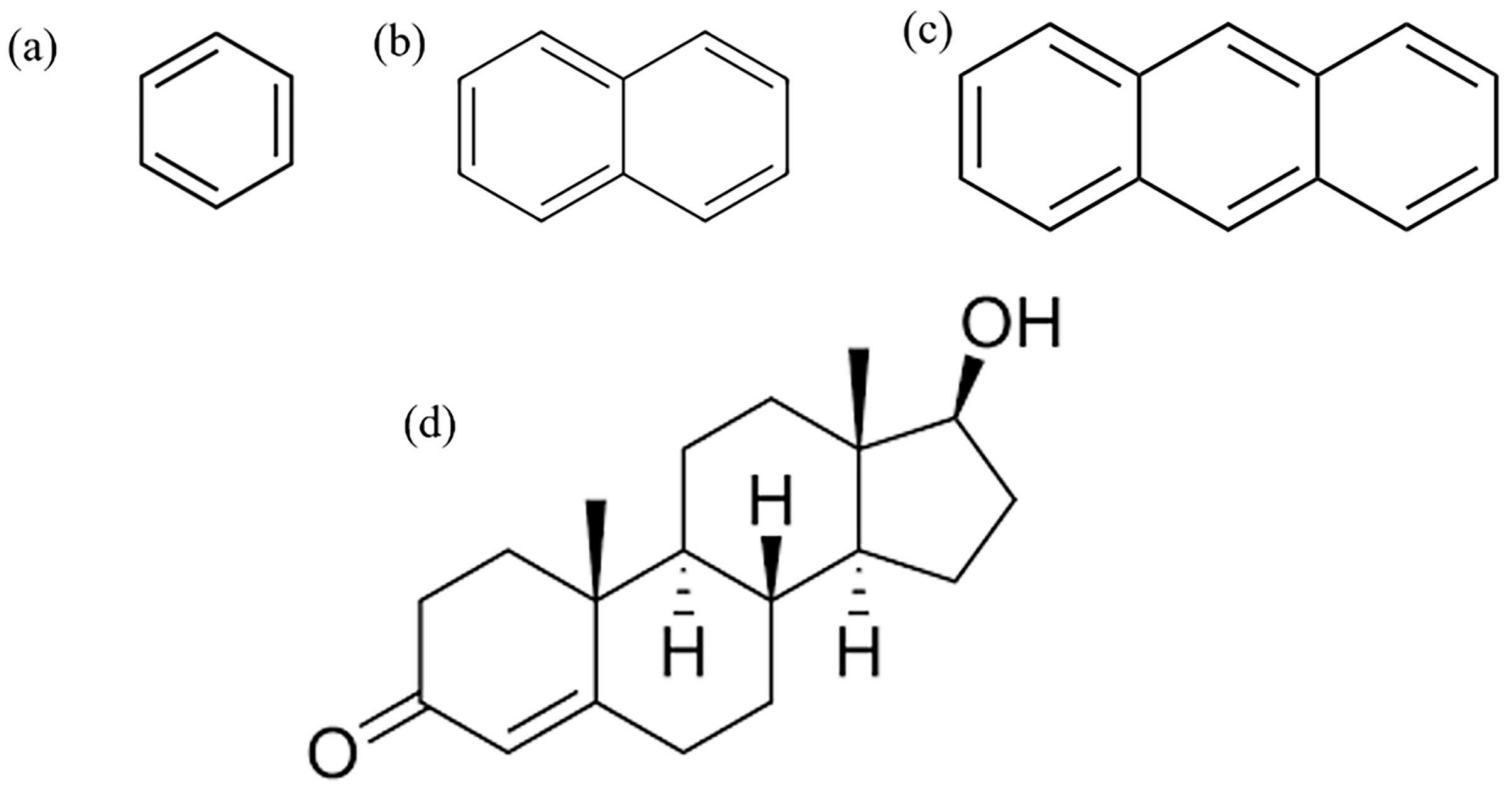
Molecular structures used in calculations: **(a)** benzene, **(b)** naphthalene, **(c)** anthracene, and **(d)** testosterone.

This study employed benzene, naphthalene, and anthracene as representative polycyclic aromatic hydrocarbons (PAHs) commonly encountered in polluted air, with testosterone serving as the target biomolecule due to its critical role in regulating physiological functions in athletes. The molecular structures of benzene, naphthalene, anthracene, and testosterone are illustrated in Figure 1. Density Functional Theory (DFT) calculations and classical all-atom Molecular Dynamics (MD) simulations were conducted to investigate molecular-level interactions between these PAHs and testosterone. Analysis of their binding behaviors and interaction energies aimed to elucidate potential hormonal disruptions caused by environmental pollutants.

### 2.2 DFT calculations

DFT calculations were performed using fully optimized structures of benzene, naphthalene, anthracene, and testosterone (Figure 1). Optimizations utilized the B3LYP functional with the 6–311 + G(d,p) basis set in the gas phase. Quantum chemical properties, including optimized geometries, molecular electrostatic potentials (MEPs), and frontier molecular orbitals, were evaluated to identify binding modes and interaction sites. Vibrational frequency analysis confirmed all stationary points as true minima. Gaussian16 software was used for geometry optimization and energy profiling, with molecular orbitals visualized via GaussView (v6.0).

### 2.3 Classical all-atom MD simulations

MD simulations employed the GROMOS force field parameters, assigning Lennard-Jones (LJ) parameters and bonded interactions sourced from the Automated Topology Builder (ATB) database. Separate simulation systems were constructed for testosterone alone and in complexes with each PAH from Figure 1. Energy minimization occurred via steepest descent for 0.1 ns at 298 K and 1 bar, followed by 1 ns equilibration runs under NVT and NPT ensembles to stabilize dimensions (10 × 10 × 10 nm^3^). Production simulations lasted 10 ns under NVT conditions at 298 K. The LINCS algorithm constrained bonds, and short-range Coulomb and LJ interactions used a 1.0 nm cutoff. Long-range electrostatics applied Particle Mesh Ewald (PME) methods, with temperature regulated by a V-rescale thermostat and pressure controlled by a Berendsen barostat. Periodic boundary conditions were implemented in all directions. Simulations were executed using GROMACS, visualized with VMD, and analyzed with SigmaPlot for free energy surfaces.

### 2.4 Pedagogical assessment methodology

To investigate the pedagogical implications of air quality on student-athlete performance, a controlled training experiment was established. Two student groups from KazNARU (n = 30 per group, males aged 18–21) participated in a semester-long physical training program. Group A trained in an urban area with high air pollution (average PM2.5 > 50 μg/m^3^), while Group B conducted identical sessions in a suburban green zone with low pollution (average PM2.5 < 10 μg/m^3^).

Both groups engaged in a standardized physical education curriculum comprising aerobic, strength, and flexibility exercises three times weekly for 90 min per session. Assessments conducted at the beginning and end of the study included pull-ups, standing long jump, and a 100 m sprint. Air-quality exposure definition & monitoring. Training sessions were classified as “polluted” when the daily mean PM_2.5_ at fixed-site monitors near the venue exceeded 50 μg·m^−3^ and “clean-air” when PM_2.5_ was <10 μg·m^−3^. PM_2.5_ was chosen as an operational index because combustion sources that emit PAHs also elevate fine-particle mass, and higher-ring PAHs commonly occur bound to PM_2.5_; therefore, contrasting PM_2.5_ regimes provide meaningful differences in particle-bound PAH exposure in the absence of speciated PAH instrumentation. We recorded temperature and relative humidity on each training day and included these (with participant covariates) in adjusted analyses; we acknowledge that PM_2.5_ is non-specific and that future work will incorporate ambient/biomarker PAH speciation.

The study adhered to ethical standards with informed consent obtained from all participants. Performance outcomes were statistically analyzed using paired t-tests for within-group changes and ANOVA for inter-group differences, considering *p*-values <0.05 statistically significant. This pedagogical assessment complemented the computational studies, providing empirical support for educational policies aimed at enhancing student health and athletic performance through improved environmental conditions.

### 2.5 Statistical analysis

Analyses were two-sided (*α* = 0.05) in R 4.x. For the three primary outcomes (100-m sprint, pull-ups, long jump), we used baseline-adjusted ANCOVA (post value as outcome; group as factor; baseline as covariate) and report mean change, adjusted group difference, Hedges’ g, and 95% CIs. Multiplicity across the three endpoints was controlled with Holm–Bonferroni (both unadjusted and adjusted p shown). Prespecified covariates were age, BMI, average nightly sleep (h), nutrition adequacy (3-level), session adherence (%), and ambient temperature/humidity; models included covariates when available. Exposure–response was examined by correlating session-averaged PM_2.5_ with individual change scores (Pearson or Spearman) with Benjamini–Hochberg FDR control. Assumptions were checked; when violated we applied log-transforms and HC3 robust standard errors. The primary ANCOVA adjusted for baseline performance; a prespecified fully adjusted model additionally included environmental covariates (daily mean temperature, relative humidity; venue-day matched) and participant covariates (age, BMI, average nightly sleep, nutrition adequacy, and session adherence) when available. Collinearity was low (all VIF < 2), model diagnostics were acceptable, and conclusions were based on the fully adjusted model.

## 3 Results

### 3.1 DFT results

#### 3.1.1 Optimized structures

**Figure 2:**
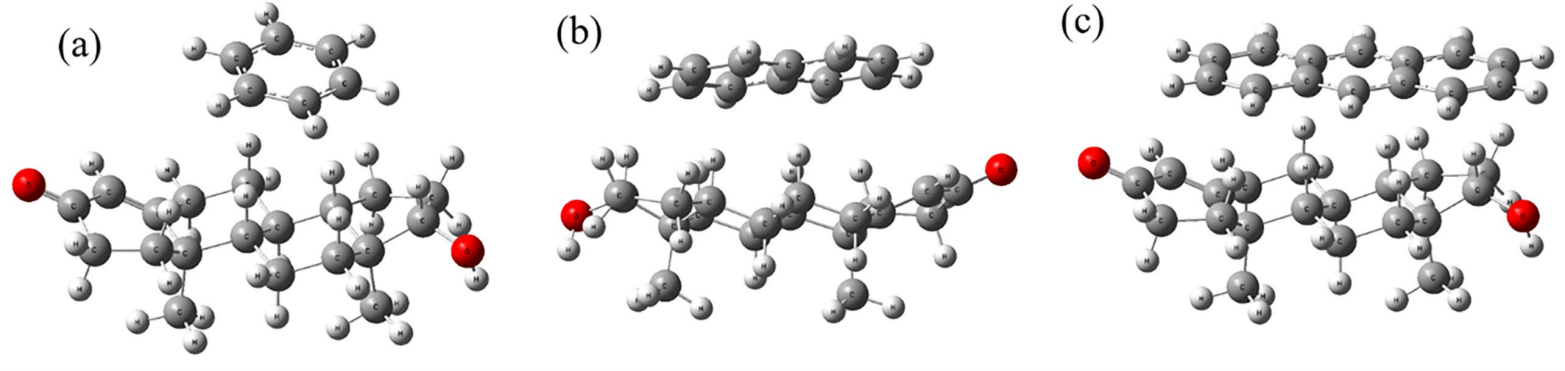
Optimized structures of testosterone interacting with **(a)** benzene, **(b)** naphthalene, and **(c)** anthracene.

Figure 2 presents the optimized geometries of testosterone complexes with benzene, naphthalene, and anthracene obtained through Density Functional Theory (DFT) calculations. In Figure 2a, benzene forms a weak *π*–π stacking interaction near testosterone’s D-ring, indicating mild van der Waals interactions. Figure 2b shows naphthalene establishing a parallel displaced stacking configuration, indicating stronger π–π interactions compared to benzene. Anthracene (Figure 2c) exhibits the strongest π–π interaction due to its extended aromatic surface, suggesting the highest binding affinity among the three PAHs.

#### 3.1.2 Molecular electrostatic potential maps

**Figure 3:**
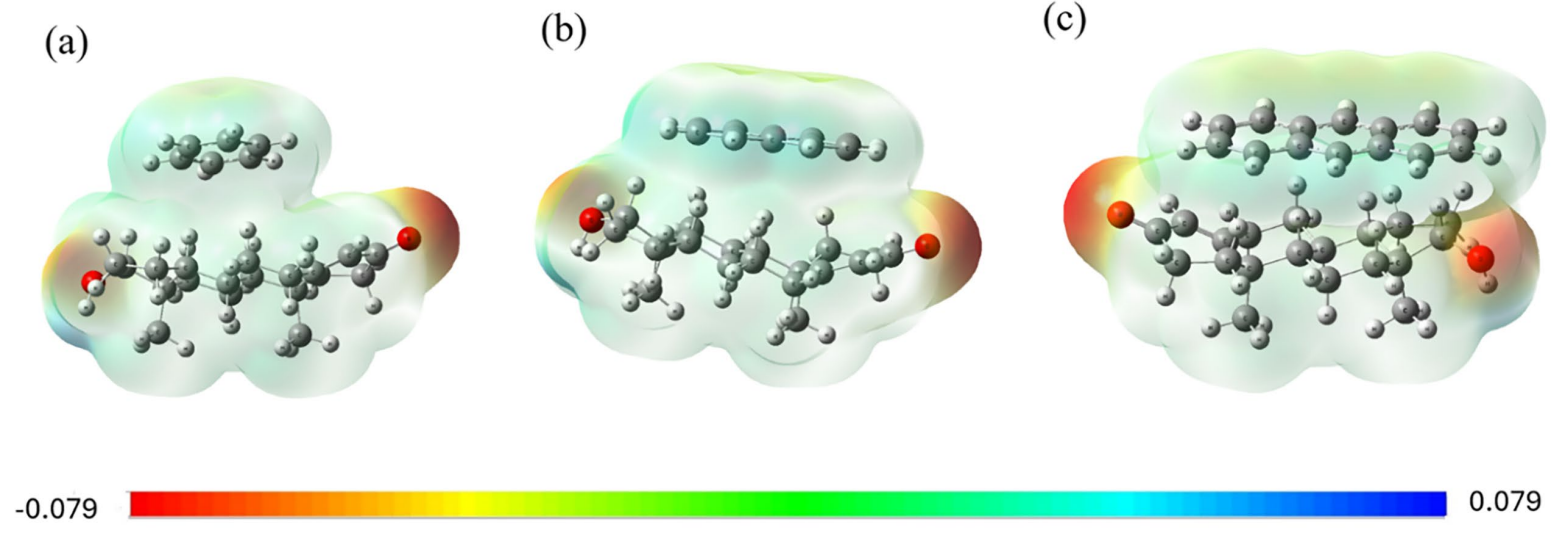
MEP surfaces for testosterone complexes with **(a)** benzene, **(b)** naphthalene, and **(c)** anthracene.

The molecular electrostatic potentials (MEPs) of the PAH-testosterone complexes are illustrated in Figure 3. The benzene-testosterone complex (Figure 3a) shows minimal polarization, indicating weak dispersion interactions. Naphthalene (Figure 3b) demonstrates increased electrostatic interactions, especially near testosterone’s hydroxyl group, suggesting stronger polarizability. Anthracene (Figure 3c) shows the strongest electrostatic complementarity, supporting a stable and significant interaction with testosterone.

#### 3.1.3 Mulliken charge distribution

**Figure 4:**
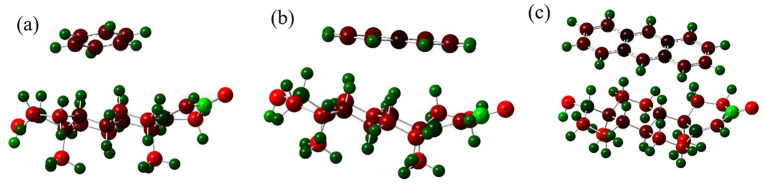
Mulliken charge distributions in complexes of testosterone with **(a)** benzene, **(b)** naphthalene, and **(c)** anthracene.

Figure 4 displays Mulliken charge distributions for each PAH-testosterone complex. Benzene (Figure 4a) exhibits minimal charge redistribution, suggesting predominantly weak van der Waals interactions. In contrast, naphthalene (Figure 4b) demonstrates more significant charge polarization, especially near testosterone’s hydroxyl region, indicating moderate electronic interactions. Anthracene (Figure 4c) shows extensive negative charge redistribution across its aromatic surface, indicating substantial electronic stabilization through *π*–π stacking and induced dipole interactions.

#### 3.1.4 Molecular orbitals

**Figure 5:**
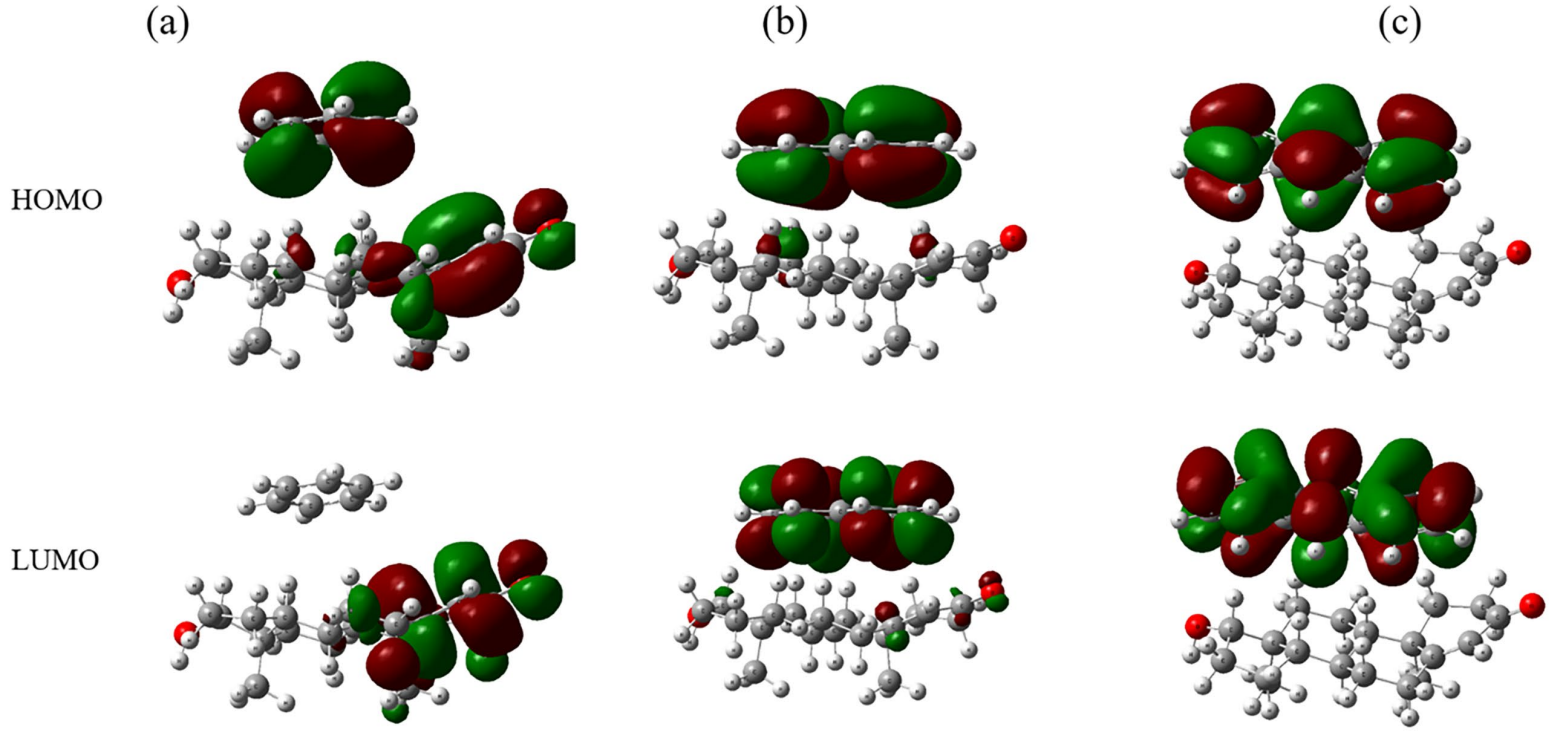
Molecular orbitals (HOMO and LUMO) for complexes of testosterone with **(a)** benzene, **(b)** naphthalene, and **(c)** anthracene.

Frontier molecular orbitals (FMOs), including HOMO and LUMO, are depicted in Figure 5. Benzene-testosterone (Figure 5a) exhibits moderate electron delocalization, with benzene acting as an electron acceptor. Naphthalene-testosterone (Figure 5b) displays increased orbital delocalization, suggesting stronger electron-donating interactions. Anthracene-testosterone (Figure 5c) shows the greatest delocalization, indicative of strong electronic interactions and potential impacts on testosterone functionality.

### 3.2 Classical all-atom MD results

#### 3.2.1 Molecular structures

**Figure 6:**
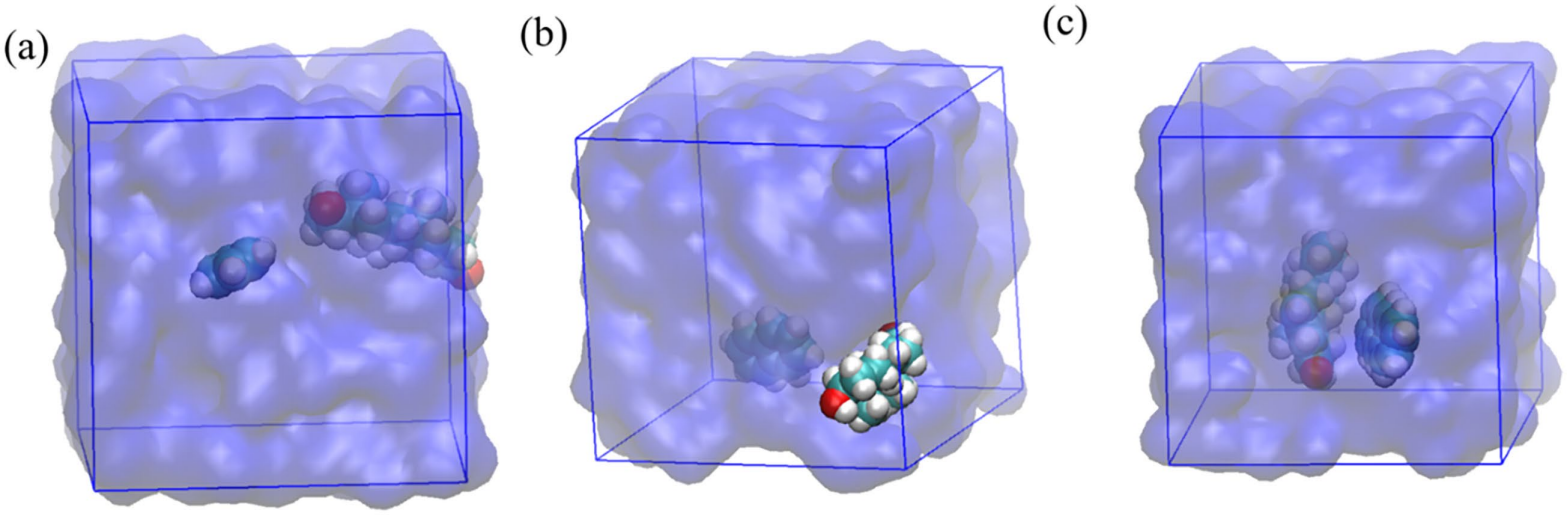
MD simulation snapshots of testosterone with **(a)** benzene, **(b)** naphthalene, and **(c)** anthracene in aqueous environments.

Molecular dynamics (MD) simulation snapshots are shown in Figure 6. Benzene (Figure 6a) demonstrates a transient, non-specific hydrophobic association with testosterone. Naphthalene (Figure 6b) exhibits more defined hydrophobic interactions, maximizing van der Waals contacts. Anthracene (Figure 6c) forms the most stable complex, strongly associating hydrophobically and suggesting optimal packing due to extended van der Waals and potential *π*–π interactions.

#### 3.2.2 Interaction energies

Calculated interaction energies (Table 1) quantify complex stability. Benzene-testosterone (+5.67 kJ/mol) indicates a slightly unfavorable interaction, suggesting minimal direct complexation. Naphthalene-testosterone (+0.16 kJ/mol) is nearly neutral, hinting at moderate interactions. Anthracene-testosterone (−4.20 kJ/mol) demonstrates a favorable interaction, indicating stable complex formation. PAHs exhibit varying solvation strengths (benzene −20.99 kJ/mol, naphthalene −15.76 kJ/mol, anthracene −16.78 kJ/mol), with reduced water affinity correlating with increased PAH size and enhanced testosterone affinity. These ring-number–dependent trends are consistent with prior DFT/MD reports in steroid–arene systems, where larger aromatic surfaces strengthen π–π/hydrophobic association in aqueous media; our magnitudes fall within the expected weak-association regime for non-covalent contacts and support the mechanistic plausibility inferred from MD and FMO analyses.

**Table 1:** Interaction energies (kJ/mol) between testosterone and PAHs.

Interaction	Benzene-testosterone	Naphthalene-testosterone	Anthracene-testosterone
PAH-testosterone	+5.67	+0.16	−4.20
PAH-water	−20.99	−15.76	−16.78
Testosterone-water	−135.52	−126.15	−126.10

### 3.3 Pedagogical performance results

Descriptive summaries (Table 2) show that, at follow-up, the fresh-air group outperformed the polluted-air group on all tests—100-m sprint 13.4 s vs. 14.5 s, pull-ups 13 vs. 9 reps, and standing long jump 225 cm vs. 210 cm—with historical cohort means provided for context. Relative to baseline, fresh-air trainees improved by −0.9 s in the sprint, +4 pull-ups, and +17 cm in the long jump, whereas changes in the polluted-air group were minimal.

**Table 2:** Comparative performance metrics.

Test	2016–2017 Avg.	2023–2024 Avg.	Fresh air group	Polluted air group	PM₂.₅ vs. Change [r (95% CI), q]*
100 m sprint (s)	14.2	14.3	13.4	14.5	+0.41 [0.17, 0.60], 0.0034
Pull-ups (reps)	11	9	13	9	−0.38 [−0.58, −0.14], 0.0041
Standing long jump (cm)	217	208	225	210	−0.35 [−0.55, −0.11], 0.0061

Between-group ANCOVA (post ~ group + baseline; covariates as available) favored the fresh-air condition for all three primary outcomes; Holm–Bonferroni–adjusted *p*-values remained significant. Covariate adjustment for temperature, humidity, and participant factors did not materially change the results (all adjusted differences remained significant with changes in point estimates <10%). Partial exposure–response correlations controlling for temperature and humidity were similar to the unadjusted analyses: sprint rₚ = +0.35 (95% CI 0.10–0.55, q = 0.007), pull-ups rₚ = −0.33 (95% CI − 0.54 to −0.09, q = 0.009), and long jump rₚ = −0.30 (95% CI − 0.51 to −0.06, q = 0.015). Consistent with these performance patterns, participants training in polluted air more frequently reported fatigue and respiratory discomfort on post-session surveys.

To assess exposure–response, session-averaged PM_2.5_ was correlated with individual change scores (see the rightmost column in Table 2). Higher PM_2.5_ was associated with smaller gains: 100-m sprint r = +0.41 (95% CI 0.17–0.60, q = 0.0034), pull-ups r = −0.38 (95% CI − 0.58 to −0.14, q = 0.0041), and long jump r = −0.35 (95% CI − 0.55 to −0.11, q = 0.0061). These findings were directionally consistent in sensitivity analyses (Spearman *ρ*; models including sleep, BMI, and adherence when available).

## 4 Discussion

The results obtained from both the computational and experimental approaches in this study provide comprehensive evidence supporting the hypothesis that polycyclic aromatic hydrocarbons (PAHs), particularly larger molecules such as anthracene, significantly interact with testosterone, potentially disrupting its physiological functions. The DFT calculations revealed a clear trend of increased interaction strength correlating with the size of the PAHs, which was evident through optimized geometries, molecular electrostatic potentials (MEPs), Mulliken charge distributions, and frontier molecular orbitals (FMOs). The progressively stronger interactions from benzene to anthracene suggest that larger PAHs pose a greater risk of hormonal disruptions due to enhanced electronic interactions and structural stability of the complexes formed with testosterone.

Molecular dynamics simulations further reinforced the computational predictions by highlighting the dynamic interactions and relative stability of the PAH-testosterone complexes within an aqueous physiological environment. Notably, anthracene displayed the strongest and most stable association with testosterone, indicating a higher propensity for biological interference. These computational insights underscore the molecular mechanisms by which environmental pollutants might adversely affect hormonal regulation in exposed individuals, particularly athletes frequently training in polluted environments.

Comparative context using existing literature only. Our semester-scale field pattern—smaller gains in sprint, pull-ups, and standing long jump under higher PM₂.₅—aligns with prior work indicating that urban air pollutants can degrade exercise responses and athlete well-being. Reviews focused on sport and active populations describe respiratory and cardiovascular strain from common pollutants and emphasize practical exposure concerns during training and events (25, 27). Within sport ecology, environmental conditions are increasingly framed as performance-relevant constraints that programs should manage alongside traditional load variables (1–3, 5, 7). Positioning PM₂.₅ as an operational exposure index is supported by reviews showing that combustion-derived higher-ring PAHs frequently occur in the fine-particle fraction and co-vary with PM₂.₅ in urban settings (10, 11). While PM₂.₅ is non-specific to PAHs, these sources justify its use for field contrasts when speciated PAH monitoring is unavailable—consistent with our design choice and limitations statement.

The endocrine rationale in our study is coherent with epidemiologic and toxicologic evidence already cited in the manuscript. Occupational and population studies report associations between PAH exposure and altered sex-steroid profiles (e.g., testosterone and estradiol), with effect directions varying by metabolite and demographic context (14, 19, 22, 23). Broader clinical and review work on androgens underscores that perturbations in androgen signaling can influence performance, recovery, and health risk in athletic settings (4, 6, 13, 15, 17, 18). Our DFT/MD finding of size-dependent noncovalent association between testosterone and larger aromatics offers a microphysical hypothesis that is compatible with these human-level observations without over-stating mechanistic causality.

Our emphasis on brief, explosive tests is consistent with sport diagnostics linking lower-limb explosive capacity to performance (8) and with evidence that short, high-intensity phases decisively contribute to outcomes in skill-power sports (9). Together, these references support using sprint and jump outcomes as environmentally sensitive markers in student-athlete cohorts.

The pedagogical component of this study complemented the computational findings by demonstrating tangible impacts of air quality on student-athlete performance. Training conducted in polluted conditions resulted in significantly reduced improvements in physical fitness metrics compared to training in fresh air. This empirical evidence confirms the detrimental effects of air pollution exposure, which could reflect impaired respiratory function, systemic inflammation, or endocrine disruptions suggested by molecular-level interactions. Furthermore, survey responses from participants training in polluted environments indicated increased fatigue and respiratory discomfort, aligning with the physiological disruptions anticipated from the literature above (25, 27).

Strengths include the integration of atomistic modeling (DFT/MD) with a controlled, short-term training comparison; prespecified analyses with confidence intervals and multiplicity control; and an exposure–response assessment linking session-level PM₂.₅ to individual change scores. Several limitations warrant caution. First, the field comparison was not randomized by venue and the sample size was moderate (male university students aged 18–21), which constrains causal inference and generalizability to other ages, sexes, or training contexts. Second, PM₂.₅ was used as an operational exposure index; while practical for field work, it is non-specific and subject to exposure misclassification relative to personal and speciated PAH measurements. Co-pollutants (e.g., NO₂, O₃) and microenvironmental factors may confound or modify associations despite covariate adjustment (10, 11). Third, some participant covariates (sleep, nutrition, adherence) were self-reported and available with missingness, introducing potential measurement error. Fourth, follow-up was limited to one academic term, capturing short-term adaptations rather than long-term training trajectories.

Computational inferences also have scope limits. DFT results depend on the chosen functional/basis [B3LYP/6–311 + G(d,p)] and gas-phase optimization, and MD simulations were short (10 ns) with force-field and solvent-model assumptions; neither framework includes macromolecular binding partners (e.g., SHBG, receptor contexts) or metabolic activation products. The PAH panel was intentionally restricted (benzene, naphthalene, anthracene) to test a size-progression hypothesis and does not cover higher-ring species. Accordingly, the modeling is hypothesis-generating and should not be over-interpreted as demonstrating endocrine disruption *in vivo*.

Because PM₂.₅ is a mixture metric and not specific to PAHs, our field contrasts cannot isolate PAH effects from co-varying pollutants; this motivates adding speciated ambient PAH measurements and/or urinary PAH metabolites in future cohorts (10, 11, 23). To address the broader limitations, future studies should employ randomized cross-over or filtration-intervention designs, incorporate personal and biomarker exposure assessment, extend computations to enhanced-sampling/free-energy methods and 4–6-ring PAHs, and include broader, mixed-sex samples with longer follow-up. In the interim, our associative findings support exposure-aware training: schedule high-intensity work on low-PM₂.₅ days and relocate key sessions indoors with MERV-13/HEPA filtration when levels are elevated; at the institutional level, integrate simple monitoring and thresholds for modifying activity—recommendations that are consistent with sport-ecology perspectives on environmental sustainability and athlete health.

## 5 Conclusion

We integrated DFT/MD modeling with a controlled training comparison to examine how PAH exposure may relate to short-term performance. While the molecular results suggest size-dependent, non-covalent association between testosterone and larger PAHs and the field data show smaller gains under higher PM_2.5_, these findings are associative and should not be over-interpreted as causal. Accordingly, the emphasis of this conclusion is on practical steps that coaches and programs can implement immediately. For coaches and athletic staff: (i) schedule high-intensity outdoor sessions when local PM₂.₅ is low (e.g., mornings/clean-air days) and substitute technique/indoor work during higher PM₂.₅ periods; (ii) relocate to indoor spaces equipped with MERV-13/HEPA filtration for hard sessions on poor-air days; (iii) avoid maximal-effort training within 24–48 h of a PM₂.₅ spike; and (iv) formalize a simple monitoring routine (assign a staff member to check PM_2.5_/AQI and adjust the daily plan), alongside athlete self-monitoring of breathlessness/fatigue to guide load adjustments.

For schools, universities, and health officials: adopt campus-level PM_2.5_ monitoring and publish real-time dashboards; define clear thresholds for modifying or postponing outdoor practices; invest in ventilation/filtration upgrades for gyms; and incorporate air-quality protocols into athletic policies and communications. To strengthen the evidence base, future work should (a) validate binding mechanisms with in-vitro assays (e.g., SPR/ITC) and endocrine reporter systems, including SHBG/testosterone contexts; (b) measure speciated ambient PAHs and/or urinary PAH metabolites alongside training outcomes; (c) use randomized cross-over or filtration-intervention designs to improve causal inference; and (d) extend computations to enhanced-sampling/free-energy methods and to 4–6-ring PAHs to test size-progression more comprehensively. Together, these steps translate the present findings into actionable practice while charting a concrete research pathway for more definitive mechanistic and policy guidance.

## Data Availability

The raw data supporting the conclusions of this article will be made available by the authors, without undue reservation.
